# Long-read sequencing reveals putatively mobilizable resistance genes and multi-drug resistance plasmids underestimated by short-read metagenomics

**DOI:** 10.71150/jm.2605007

**Published:** 2026-08-31

**Authors:** Dabin Jeon, Tatsuya Unno

**Affiliations:** 1 CJ Bioscience Inc., Seoul 04527, Republic of Korea; 2 Department of Biological Sciences and Biotechnology, Chungbuk National University, Cheongju 28644, Republic of Korea

**Keywords:** metagenomics, antimicrobial resistance, mobilome, plasmid, long-read sequencing, hybrid assembly

## Abstract

While shotgun metagenomics is often used to profile antibiotic resistome in gut microbial communities, few studies have investigated if the choice of sequencing platform and assembly strategy affect what mobile genetic elements and antimicrobial resistance genes are recovered. In this study, we compared three platforms (Illumina, Oxford Nanopore, and PacBio HiFi) and seven assembly strategies on gut metagenomes from cattle, pig, and human as case studies. Long-read assemblies recovered 5- to 7-fold more plasmid sequence than Illumina in cattle and pig (mean 17.0 Mb vs. 3.1 Mb), while Illumina performed comparably in the less diverse human gut where high per-species coverage enabled effective short-read plasmid assembly. Long reads also detected more resistance genes on plasmid contigs. Hybrid assembly results depended on the algorithm: scaffolding-based OPERA-MS preserved long-read contiguity and recovered more plasmid-borne resistance genes, while the short-read-centric metaSPAdes hybrid mode produced fragmented assemblies. After collapsing haplotype redundancy, PacBio HiFi identified 2 and 49 unique multi-drug resistance plasmid lineages in cattle and pig, respectively. On the other hand, only 2 and 4 were identified from Illumina. Long reads also placed far more ARGs in a putative mobilization context (50–73%) compared to 14–21% for short reads. Platform and assembly strategy are thus key variables in mobilome and resistome characterization and should be accounted for in antimicrobial resistance surveillance.

## Introduction

Shotgun metagenomics is now a standard approach for profiling microbial communities in environments ranging from the human gut to agricultural settings (Quince et al., 2017). It allows simultaneous assessment of community composition, functional potential, and clinically relevant genes such as antimicrobial resistance (AMR) determinants (Hendriksen et al., 2019). What metagenomics can recover, though, depends heavily on the sequencing platform used.

Illumina short-read sequencing has dominated the field owing to high per-base accuracy and low cost, but short read lengths limit assembly contiguity. This makes it hard to resolve repeats, link genes to their genomic context, or fully reconstruct mobile genetic elements (MGEs) (Arredondo-Alonso et al., 2017). Long-read platforms such as Oxford Nanopore Technologies (ONT) and Pacific Biosciences (PacBio) HiFi generate much longer reads (> 10 kb depending on library preparation), and can produce far more contiguous metagenome assemblies (Bickhart et al., 2022; Moss et al., 2020). Hybrid assembly, on the other hand, combines the base-level accuracy of short reads with the contiguity of long reads to yield assemblies that are accurate and contiguous (Wick et al., 2017). While metaSPAdes builds a de Bruijn graph from short reads and threads long reads through it to resolve ambiguities (Nurk et al., 2017), OPERA-MS scaffolds a short-read assembly backbone using long reads for gap-filling (Bertrand et al., 2019).

Mobilome characterization is especially sensitive to contiguity (Maguire et al., 2020). Plasmids are the main vehicles for horizontal AMR gene transfer, and knowing whether a resistance gene sits on a mobile element or the chromosome is key to predicting resistance spread (Partridge et al., 2018). Characterization of plasmids, especially the ones with multi-drug resistance (MDR) genes, is extremely important for One Health surveillance because a single transfer event can disseminate multiple resistance phenotypes at once (Carattoli, 2013). Beyond plasmid identification, capturing the flanking mobilization machinery such as insertion sequences and transposons around ARGs is critical for assessing horizontal gene transfer potential. Short-read contigs are often too fragmented to preserve this context.

Recent platform comparisons have addressed parts of this question. Eisenhofer et al. (2024) provided a valuable comparison of short-read, PacBio HiFi, and hybrid strategies for MAG recovery from mouse gut, demonstrating the benefits of long reads for genome completeness; however, their study did not examine the mobilome or resistome and used laboratory mice in a controlled environment, limiting the relevance of their findings to AMR surveillance. Lorenzin and Carlin (2025) offered a useful review of long-read versus short-read metagenomics for respiratory diagnostics, though their focus was on pathogen detection rather than assembly-based mobilome analysis. To date, no study has included all three major platforms (Illumina, ONT, PacBio HiFi), tested multiple hybrid algorithms, and evaluated how platform choice affects plasmid-borne AMR gene detection or MDR plasmid recovery.

Here, we compared all three platforms on the same DNA extracts from cattle, pig, and human gut metagenomes as case studies, using seven assembly strategies that include two hybrid approaches. Our focus was on how platform and assembly choices affect plasmid recovery, plasmid-borne AMR gene detection, and MDR plasmid identification, directly relevant to AMR surveillance.

## Materials and Methods

### Sample collection and DNA extraction

Fecal samples were collected from cattle, pig, and human. Animal fecal samples were collected from freshly voided feces without direct handling of or contact with the animals. The human sample was obtained from a healthy adult volunteer with no comorbidities and no recent use of antibiotics or probiotics. These three taxonomically and ecologically distinct gut metagenomes serve as case studies for the platform comparison. High-molecular-weight genomic DNA was extracted using the Wizard HMW DNA Extraction Kit (Promega, USA) according to the manufacturer's instructions. This kit uses chemical lysis without mechanical disruption (e.g., bead-beating), which may underrepresent Gram-positive organisms with thick peptidoglycan cell walls; however, this bias affects all platforms equally since the same extraction was used. DNA concentration was measured using the Qubit dsDNA HS Assay Kit (Invitrogen, USA), and fragment size distribution was assessed using the Bioanalyzer system with the DNA 12000 Kit (Agilent Technologies, USA). Only DNA samples with an input amount of at least 3 μg and an OD_260/280_ ratio of 1.8–2.0 were used for library preparation. The same DNA extraction was used for all three sequencing platforms to minimize extraction-related biases.

### Library preparation and sequencing

Library preparation and sequencing were performed by CJ Bioscience Inc. (Korea). Each sample was sequenced on all three platforms, yielding nine datasets in total (3 samples × 3 platforms).

For PacBio HiFi sequencing, genomic DNA underwent damage repair, end repair, and A-tailing using the SMRTbell Prep Kit 3.0 (Pacific Biosciences, USA). SMRTbell adapters were ligated, followed by exonuclease treatment to remove unligated fragments. Templates were purified using AMPure PB magnetic beads at approximately 0.45× bead volume to enrich for large fragments. Long-read shotgun metagenomic sequencing was performed on the PacBio Revio System using diffusion loading into zero-mode waveguides (ZMWs).

For ONT sequencing, genomic DNA was fragmented to approximately 10 kb using a FastPrep-24 5G instrument (MP Biomedicals, USA), and fragment size distribution was verified on the Bioanalyzer system with the DNA 12000 Kit (Agilent Technologies, USA). Observed mean read lengths (4.6–5.5 kb) were shorter than the 10 kb fragmentation target because subsequent library preparation steps (end-repair, adapter ligation, AMPure bead cleanup) and flow cell loading preferentially retain shorter fragments, and the FastPrep produces a broad size distribution around the target. Libraries were constructed using the Native Barcoding Kit (Oxford Nanopore Technologies, UK) according to the manufacturer’s protocol. Shotgun metagenomic sequencing was performed on the ONT GridION system using an R10.4.1 flow cell. Basecalling was performed using Dorado with the super-accuracy (SUP) basecalling model v4.3.0 at 400 bp, with built-in adapter trimming enabled.

For Illumina sequencing, libraries were constructed using the NEBNext^®^ Ultra^TM^ II FS DNA Library Prep Kit (New England Biolabs, USA) according to the manufacturer's protocol. Shotgun metagenomic sequencing was performed on the Illumina NovaSeq 6000 system (Illumina, USA) with paired-end 2 × 150 bp reads using the NovaSeq 6000 S2 Reagent Kit v1.5 (300 cycles, XP workflow).

### Read processing and assembly

Illumina reads were trimmed and quality-filtered using fastp v0.23.4 (Chen et al., 2018) with a minimum quality score of 20 and minimum length of 50 bp. ONT reads were quality-filtered with Chopper v0.7 (De Coster et al., 2018) using a minimum quality score of 10 and minimum read length of 1,000 bp; adapter trimming was handled by Dorado’s built-in function during basecalling. PacBio HiFi reads were length-filtered (minimum 1,000 bp) but otherwise used without additional trimming. Host-derived reads were removed by mapping Illumina reads against host reference genomes using Bowtie2 (Langmead and Salzberg, 2012) and long reads using minimap2 (Li, 2018). Seven assembly strategies were applied to each sample, producing 21 assemblies in total (Table S2): MEGAHIT v1.2.9 (Li et al., 2016) for Illumina; metaFlye v2.9 with the `--meta` flag (Kolmogorov et al., 2020) for ONT; hifiasm-meta (Feng et al., 2022) for PacBio; metaSPAdes v4.0.0 (Nurk et al., 2017) for hybrid Illumina+ONT and Illumina+PacBio; and OPERA-MS (Bertrand et al., 2019) using MEGAHIT contigs as backbone with long-read scaffolding for both ONT and PacBio. Assembly quality was assessed using QUAST (Gurevich et al., 2013).

### Taxonomic and functional annotation

Read-based taxonomic profiling was performed using Kraken2 (Wood et al., 2019) with the standard database, followed by Bracken (Lu et al., 2017) for species-level abundance estimation on quality-controlled, host-removed reads. Open reading frames (ORFs) were predicted and annotated using Prokka v1.14.6 (Seemann, 2014) with the `--metagenome` flag for all 21 assemblies.

### Mobilome and resistome analysis

Contig classification into chromosome, plasmid, and virus categories was performed using geNomad v1.11.2 (Camargo et al., 2024) with the `end-to-end` subcommand and default parameters, including a classification score threshold of 0.7 for both plasmid and virus assignments. Total resistome analysis was performed by running AMRFinderPlus v3.12 (Feldgarden et al., 2021) with the `--plus` flag on full assembly contigs for all seven assembly strategies; plasmid-borne AMR genes were identified separately by running AMRFinderPlus on geNomad-classified plasmid contigs. ARG density was calculated as unique ARGs per 100 Mb of total assembled sequence; an additional normalization (ARGs/1,000 predicted CDS) is provided in Table S11 as a complementary metric less sensitive to assembly size inflation. Note that AMRFinderPlus `--plus` reports both antibiotic resistance genes and metal/biocide resistance genes (e.g., copper, arsenic, silver, nickel); these are distinguished in the results. MDR plasmids were defined as individual plasmid contigs carrying AMR genes belonging to two or more drug classes. This contig-level definition inherently favors more contiguous assemblies, as a physically MDR plasmid fragmented across multiple short contigs cannot be classified as MDR. For hifiasm-meta assemblies, which produce multiple haplotype contigs per assembly subgraph representing strain-level variants, ARG counts and MDR classifications were computed at the subgraph level to avoid inflation from haplotype redundancy. Specifically, all contigs sharing the same hifiasm-meta subgraph identifier were treated as haplotype variants of a single genomic locus; the union of ARG annotations across haplotype contigs within each subgraph was taken as the representative ARG profile for that plasmid lineage. A "unique plasmid lineage" thus corresponds to a single hifiasm-meta subgraph for PacBio assemblies. For cross-platform comparisons, plasmid contigs were clustered at 90% sequence identity using MMseqs2 easy-cluster (Table S4). To assess the putative mobilization context of ARGs, Prokka GFF annotations were parsed for mobile genetic element (MGE) markers — insertion sequences (IS), transposons (Tn), integrases/recombinases (Int), conjugation machinery (Conj), and phage elements — co-located on the same contig as AMRFinderPlus-identified ARGs across all 21 assemblies. The specific keyword list used to classify each MGE category from Prokka product annotations is provided in Table S12.

### Metagenome-assembled genome recovery

Two binning strategies were compared. For short-read (MEGAHIT) and hybrid assemblies (metaSPAdes, OPERA-MS), MetaBat2 (Kang et al., 2019) was run on Illumina read alignments generated with Bowtie2 and summarized with `jgi_summarize_bam_depths`, followed by bin refinement with DAS Tool (Sieber et al., 2018). For long-read assemblies (metaFlye, hifiasm-meta), SemiBin2 (Pan et al., 2023) was run in long-read mode (`single_easy_bin --sequencing-type long_read`) using native long-read coverage from minimap2 alignments (`map-ont` for ONT reads, `map-hifi` for PacBio HiFi reads). This split was motivated by a preliminary comparison showing that MetaBat2 with Illumina coverage produced a high proportion of low-quality bins from long-read assemblies, whereas SemiBin2 with native long-read coverage recovered 1.4–2.2× more HQ+MQ MAGs. Bin quality was assessed using CheckM2 (Chklovski et al., 2023), with high-quality (HQ) bins defined as completeness ≥ 90% and contamination < 5%, and medium-quality (MQ) bins as completeness ≥ 50% and contamination < 10%. Taxonomic classification of HQ+MQ bins was performed using GTDB-Tk v2.6.1 (Chaumeil et al., 2022) with the GTDB r226 database.

### Downsampling analysis

To test whether differences in sequencing depth between hosts confounded platform comparisons, cattle and pig long reads were subsampled to match human long-read depth using seqtk sample (v1.4). ONT reads were subsampled from 10.07/9.70 Gb to 6.02 Gb, and PacBio reads from 10.16/11.77 Gb to 7.39 Gb. Subsampled reads were reassembled using the same pipelines (metaFlye for ONT, hifiasm-meta for PacBio) and classified with geNomad under identical parameters. Plasmid content was compared between full-depth and subsampled assemblies.

### External validation with independent human gut metagenomes

To test whether the observed host-dependent platform effects generalize beyond our single human sample, three independent human gut metagenome samples from Chen et al. (2022) (BioProject PRJNA820119) were obtained from the NCBI Sequence Read Archive. These samples represent healthy adults from a cross-sectional cohort sequenced with both Illumina NovaSeq 6000 (8.6–11.7 Gb) and ONT PromethION (4.6–10.8 Gb) from the same DNA extraction. Illumina reads were assembled with MEGAHIT and ONT reads with metaFlye using the same parameters as the primary analysis. Plasmid contigs were identified with geNomad v1.11.2 under identical settings.

### Statistical analysis

Platform effects on assembly metrics were assessed using the Friedman test, a non-parametric repeated-measures test appropriate for the blocked design (three case studies × three single-platform assembly strategies). Three samples represent the minimum for this test, and results should be interpreted with caution; validation with larger cohorts is warranted. Plasmid content comparisons (Fig. 2) were assessed using Friedman tests with post-hoc Nemenyi tests. Community structure differences across hosts and platforms were tested with ANOSIM on Bray-Curtis distances. Significance was evaluated at α = 0.05. All statistical analyses were performed in Python using SciPy v1.14.

## Results

### Sequencing yield and assembly quality

All three platforms produced sufficient data for metagenomic assembly across the three samples (Table S1). Illumina generated the most reads (> 55 million reads), followed by ONT and PacBio HiFi (1.0–2.1 millions reads). Mean read lengths of ONT and PacBio HiFi ranged from 4.6 kb to 5.5 kb and 6.2 kb to 6.8 kb, respectively.

Assembly statistics were summarized in Table S2. Long-read assemblies (metaFlye, hifiasm-meta) achieved N50 values ranging from 30 kb to 69 kb, which is 7- to 13-fold higher than Illumina MEGAHIT. metaSPAdes hybrid assemblies were highly fragmented (49,285–103,642 contigs) with N50 values (5.5–9.5 kb) barely above Illumina-only, despite incorporating long reads. OPERA-MS hybrid assemblies, on the other hand, were far more contiguous (N50 24.2–41.9 kb, only 26,273–30,822 contigs), yet lower than long-read-only assembly. The scaffolding approach thus preserves long-read contiguity better than the de Bruijn graph approach, though it does not fully match single-platform long-read performance.

### Taxonomic profiling across platforms

Kraken2/Bracken profiling showed broadly similar phylum-level community compositions across platforms for each sample (Fig. 1A). Dominant phyla shifted somewhat between platforms: for example, Pseudomonadota dominated Illumina profiles while Bacillota dominated long-read profiles in cattle. Other than this, similar patterns were observed across platforms. NMDS ordination showed that samples clustered primarily by host species rather than sequencing platform (Fig. 1B; stress = 0.417). The high stress value exceeds the commonly accepted threshold for reliable two-dimensional ordination (< 0.2), so the spatial arrangement should be interpreted with caution; however, ANOSIM confirmed a strong host effect (R = 1.00, *p* = 0.004) and no significant platform effect (R = −0.23, *p* = 0.872), indicating that platform choice has a limited effect on overall community composition despite the read-level differences noted above. On the other hand, Illumina detected 15–50% more species and 20–50% more genera than long-read platforms across all samples (11,448–13,383 species for Illumina vs. 5,808–11,469 for PacBio HiFi; Table S3), likely because its higher read throughput increases the chance of sampling rare taxa.

### Plasmid and viral sequence recovery

Assembly strategy had a large effect on mobilome recovery (Fig. 2). Friedman tests across the three hosts showed a trend toward significant differences among the seven strategies (*χ*² = 10.14, *p* = 0.119) and among the three single-platform strategies (*χ*² = 2.67, *p* = 0.264); the lack of significance reflects the limited statistical power with n = 3 case studies rather than absence of biological effect, as the 5- to 7-fold differences described below are consistent across hosts. In both cattle and pig, long-read assemblies recovered 5- to 7-fold more plasmid sequence than Illumina. MEGAHIT recovered only 2.9–3.2 Mb of plasmid content across the two livestock samples, while ONT metaFlye and PacBio hifiasm-meta recovered 12–22 Mb each. This pattern was different in the human sample, where Illumina plasmid content was comparable to those of ONT and PacBio.

The platform gap was even wider for viral content (up to 8-fold in livestock). Hybrid strategies fell in between. OPERA-MS recovered 5.5–6.0 Mb of plasmid content in cattle and pig, which is more than Illumina-only but less than long-read-only assemblies. metaSPAdes hybrid recovered slightly more plasmid sequence (7.9–9.5 Mb) but spread across far more fragmented contigs.

The human sample had the lowest assembled content across all categories regardless of platform or assembly strategy (Fig. 2), and the platform gap between Illumina and long reads was substantially smaller than in livestock.

Plasmid contig lengths also differed (Fig. S1). PacBio hifiasm produced the longest plasmid contigs (median 3–5× longer than MEGAHIT), while metaSPAdes hybrid contigs were nearly as short as MEGAHIT despite incorporating long reads.

### Total resistome

We compared total ARG detection (chromosomal + plasmid) across all seven strategies (Fig. 3). After normalizing by assembly size, Illumina MEGAHIT and OPERA-MS hybrid had the highest ARG density (15–49 unique genes/100 Mb), while PacBio hifiasm had the lowest (4–36/100 Mb; Fig. 3A). The higher raw ARG counts in long-read assemblies thus largely reflect their larger assembly sizes, not better detection sensitivity. ARG density varied more across strategies in cattle (4–17/100 Mb) than in human (36–49/100 Mb).

The strategies differed more in how they attributed ARGs to mobile elements (Fig. 3B). In cattle and pig, ONT attributed 51–68% of detected ARGs to plasmid contigs versus only 22–28% for Illumina. metaSPAdes hybrid had the lowest plasmid attribution in livestock (20–31%), even below Illumina-only, its fragmented contigs fail geNomad plasmid classification. OPERA-MS did better (27–44%), approaching long-read levels. In human, the pattern was reversed: Illumina-based methods attributed the most ARGs to plasmids (MEGAHIT 60%, OPERA-MS+PB 66%).

### Plasmid-borne resistome

The distribution of plasmid-borne ARGs across drug classes differed markedly by assembly strategy (Fig. 4). Long-read assemblies (metaFlye and hifiasm) detected ARGs across nearly all drug classes, while MEGAHIT detected only a subset. The difference was most striking for metal resistance genes (copper, nickel, arsenic, and silver), which are distinct from antibiotic resistance genes but relevant to co-selection dynamics, and were detected almost exclusively by long-read platforms in livestock. metaSPAdes hybrid was notably sparse, comparable to or worse than MEGAHIT for several drug classes, while OPERA-MS showed intermediate detection.

Quantitatively, long-read assemblies found more unique AMR genes and drug classes on plasmid contigs than short-read or hybrid approaches (Table 1). ONT metaFlye detected 49 unique genes across 22 drug classes in cattle, while MEGAHIT found only 8 genes across 5 classes from the same sample. PacBio hifiasm consistently detected the most drug classes across all samples. Because hifiasm produces haplotype-variant contigs for strain variants within the same assembly subgraph, we collapsed redundant hits: 214 ARG hits remained across 121 unique plasmid lineages, showing that common genes like *tet*(Q) and *tet*(W) are independently carried by many distinct plasmids.

MDR plasmid detection showed the largest platform gap (Table 1). All PacBio statistics are reported at the subgraph level to avoid haplotype inflation. PacBio hifiasm consistently identified the most MDR plasmid lineages (each a unique subgraph carrying ARGs from ≥ 2 drug classes), followed by ONT metaFlye, while MEGAHIT detected the fewest — up to 12-fold difference between PacBio and Illumina. This difference partly reflects the contig-level MDR definition: short-read contigs may be too short to capture multiple co-located resistance genes on a single plasmid, so a physically MDR plasmid fragmented across multiple MEGAHIT contigs would not be classified as MDR. To assess this bias, we aligned MEGAHIT plasmid contigs against PacBio subgraph sequences using minimap2 and confirmed that many single-class MEGAHIT contigs map to PacBio MDR plasmid subgraphs (Table S13), indicating that the platform gap reflects genuine fragmentation-driven underdetection rather than solely a definitional artifact. Among hybrid strategies, OPERA-MS outperformed metaSPAdes hybrid, which detected the fewest MDR plasmids likely because its fragmented contigs split co-located resistance genes across separate fragments.

The human pattern for ARG detection mirrored the mobilome results: Illumina detected as many or more unique plasmid-borne genes (56) as PacBio hifiasm-meta (45). In this sample, 78% of Illumina plasmid contigs were under 10 kb (Fig. S1), and Illumina recovered more total plasmid sequence (12.5 Mb) than PacBio (8.5 Mb). Clustering plasmid contigs at 90% identity (MMseqs2) showed that Illumina-only clusters dominated in human (53% of all clusters vs. 21–24% in livestock; Table S4), while long-read-only clusters dominated in cattle and pig — these are diverse, low-abundance plasmid lineages that require long-read contiguity to assemble.

### ARG mobilization context

To assess whether detected ARGs reside in a putative mobilization context, we identified ARG-carrying contigs co-located with MGE markers — IS elements, transposons, integrases, conjugation machinery, and phage elements — across all 21 assemblies (Fig. 5, Table S5). Long-read assemblies detected 3- to 9-fold more conjugation genes on plasmid contigs than MEGAHIT (Table S6), and most ARG-carrying plasmid contigs in long-read assemblies were also conjugative (e.g., 29 of 37 for metaFlye in cattle). PacBio hifiasm consistently placed the highest proportion of ARG contigs in a putative mobilization context (50–73%), compared to only 14–21% for Illumina MEGAHIT (Fig. 5A). The absolute MGE counts for hifiasm are inflated by haplotype redundancy — e.g., 701 ARG contigs from only 85 unique gene symbols, with *tet*(W) alone appearing on 122 haplotype-variant contigs — but the putatively mobilizable percentage remains valid as it reflects the proportion of contigs with MGE context regardless of redundancy. Co-localization on the same contig suggests a mobilization context but does not prove actual transferability; experimental validation (e.g., conjugation assays) would be required to confirm mobility. MEGAHIT detected zero IS elements on ARG contigs across all three samples, because its short contigs (~3–5 kb median) capture ARGs but not the flanking IS elements (typically 700–2,500 bp) that mediate mobilization between replicons.

metaSPAdes hybrid detected only 10–40% of ARGs in mobilization context and missed conjugation-associated ARGs entirely in most samples. OPERA-MS did better (28–47%), in line with its higher contiguity. Conjugation and phage markers on ARG contigs were almost exclusive to long-read assemblies: hifiasm found 4–61 conjugation-associated ARG contigs per sample versus 0–1 for MEGAHIT and 0–2 for metaSPAdes hybrid (Fig. 5B, Table S5). Short-read approaches thus miss not only plasmid-borne ARGs but also the putative mobilization machinery flanking detected ARGs.

### Assembly accuracy and its impact on ARG and plasmid detection

Assembly accuracy affected downstream analyses (Table S9). Friedman tests confirmed significant platform effects for CDS, rRNA, and tRNA densities (all *χ*² = 6.00, *p* = 0.050). ONT metaFlye assemblies had inflated CDS density (~1,170 CDS/Mb vs. ~980 for PacBio and Illumina), likely from Prodigal overcalling fragmented ORFs around indel errors, while PacBio hifiasm-meta recovered the most rRNA and tRNA genes per Mb.

These differences carried over to ARG detection. AMRFinderPlus found ARGs on a higher fraction of PacBio plasmid contigs (4.2–15.8%) than ONT (2.4–3.3%). ONT assemblies produced INTERNAL_STOP hits for clinically important ARGs (*mef*(A), *erm*(B), *bla*OXA, *tet*(X2), *fos*A7) — premature stop codons likely caused by residual indel errors. INTERNAL_STOP hits were not exclusive to ONT: PacBio assemblies produced 46 across three samples (including 39 in pig) and Illumina produced 5, compared to 15 for ONT. AMRFinderPlus reports these hits but does not exclude them from output, meaning they could inflate ARG counts in automated surveillance pipelines. No polishing step (e.g., Medaka) was applied to ONT assemblies in this study, which may have contributed to ONT-specific artifacts; future work should evaluate the effect of polishing on INTERNAL_STOP reduction. Accuracy affected plasmid completeness too: ONT metaFlye had the lowest circular plasmid recovery rate (0.1–0.4% DTR/ITR) despite having the most total plasmid content, because residual errors at contig junctions disrupt terminal repeat recognition. OPERA-MS hybrid had the highest circularity in cattle and human (3.5–3.7% and 1.9%), combining Illumina base accuracy with long-read scaffolding. PacBio hifiasm led in pig (2.6%, 27 DTR plasmids). Even MEGAHIT recovered more circular plasmids than ONT metaFlye (0.6–2.7%) — for plasmid closure, base accuracy matters more than read length.

### MAG recovery and quality

Binning tool choice substantially affected MAG recovery from long-read assemblies (Table 2). When MetaBat2 was applied with Illumina coverage, hifiasm produced the highest proportion of LQ bins in livestock (41–57%), suggesting poor compatibility between Illumina coverage profiles and haplotype-resolved contigs. SemiBin2 with native long-read coverage recovered 1.4–2.2× more HQ+MQ MAGs across all six long-read assemblies (e.g., 40 to 87 for cattle hifiasm, 46 to 81 for pig metaFlye), confirming that binning tool selection is critical for long-read metagenomics. hifiasm assemblies remained harder to bin than metaFlye regardless of tool, indicating that haplotype redundancy is an inherent challenge for current binning algorithms. Based on this comparison, we used SemiBin2 for long-read assemblies and MetaBat2 for short-read and hybrid assemblies in all subsequent analyses.

HQ+MQ MAGs were taxonomically classified with GTDB-Tk (Fig. 6, Table S8). The long-read assemblies, now properly binned, recovered the broadest genus diversity: metaFlye captured 39–67 unique genera per sample and hifiasm 45–67, compared to 12–26 for MEGAHIT. Only 4–15 genera (4–16% of total) were shared by all seven strategies, while 12–34 genera were exclusive to a single strategy. Total genus counts across all strategies were 96 (cattle), 80 (pig), and 54 (human).

## Discussion

This study shows that sequencing platform and assembly strategy are major determinants of what metagenomics can reveal about the mobilome and resistome. The differences go beyond quantity: long-read platforms detected MDR plasmids and gene co-localization patterns that short-read sequencing simply could not capture. Previous comparisons have made important contributions to MAG recovery (Eisenhofer et al., 2024) and pathogen detection (Lorenzin and Carlin, 2025), but none have compared all three major platforms with multiple hybrid algorithms on the same biological samples while focusing on mobilome and resistome recovery.

### Long reads are essential for mobilome characterization

Long-read platforms recovered more plasmid content across all three hosts, consistent with findings in isolate genomics (Arredondo-Alonso et al., 2017). The 12-fold MDR plasmid gap between PacBio and Illumina means short reads underestimate how many resistance genes sit on mobile elements. The putative mobilization context analysis reinforced this — only 14–21% of ARG contigs in Illumina assemblies co-located with MGE markers versus 50–73% for PacBio hifiasm, suggesting that ARGs appearing chromosomal in short-read data may actually be flanked by mobilization machinery, though experimental validation would be needed to confirm actual transferability.

The human sample was an exception. Validation with three independent human gut metagenomes from a cross-sectional cohort (Chen et al., 2022) confirmed that this is not an outlier: ONT/Illumina plasmid content ratios ranged from 0.68× to 1.52× (mean ~1.0×) across three external samples, consistent with the 1.0× ratio in our human sample, versus 5.4–5.6× for cattle and pig (Table S10). Per-Gb normalization, downsampling (Table S7), clustering, and external validation all point to a biological cause. Two factors may explain this pattern. First, low microbial diversity in the human gut (Fig. S2; Song and Unno, 2024) means higher per-species coverage, and Illumina's large read count (~55 million at 8 Gb vs. ONT's ~1.5 million) becomes an advantage when each species already has deep coverage — read breadth outweighs contiguity. Second, we hypothesize that livestock exposure to antimicrobials and heavy metals may drive co-selection of metal and antibiotic resistance on conjugative plasmids (Baker-Austin et al., 2006), creating a diverse plasmid pool with many conjugative elements at low frequency (Zhu et al., 2013), while healthy human donors carry fewer but more dominant plasmid lineages. Our study design does not directly test this co-selection mechanism, and targeted experimental studies would be needed to confirm it. The long-read advantage thus scales with community plasmid diversity; where small, abundant plasmids dominate, short reads perform comparably.

### PacBio HiFi vs. ONT for metagenomics

Both long-read platforms outperformed Illumina for mobilome recovery, but they have different strengths. PacBio HiFi detected three times more MDR plasmid lineages than ONT in pig despite producing fewer total contigs. This reflects longer plasmid contigs (median 18.2 kb vs. 9.8 kb), higher accuracy (> Q20 vs. ~Q10–15) that improves ARG detection, and better contiguity for reconstructing full gene cassette architecture on large conjugative MDR plasmids (mean 2.8 drug classes per contig, max 10 vs. ONT mean 2.3, max 3). ONT's ultra-long reads, on the other hand, may capture broader resistance gene diversity even at lower accuracy.

The INTERNAL_STOP artifacts found in ARG sequences (see Results) show that accuracy affects not just detection but functional interpretation. While ONT assemblies produced INTERNAL_STOP hits for clinically important ARGs, these artifacts were not exclusive to ONT — PacBio assemblies also produced INTERNAL_STOP hits (46 total vs. 15 for ONT), likely from genuine pseudogenes or assembly errors in repetitive regions. Surveillance pipelines should filter or flag INTERNAL_STOP annotations regardless of platform, and ONT-based workflows should incorporate assembly polishing (e.g., Medaka) to minimize false truncation calls. ONT’s error profile could also compromise detection of point-mutation-mediated resistance (e.g., *gyrA* mutations in fluoroquinolone resistance), though such mutations are typically chromosomal and were not observed here.

### Hybrid assembly: Algorithm matters more than data combination

Not all hybrid approaches are equal — the algorithm matters more than simply combining two data types. metaSPAdes hybrid is fundamentally a short-read assembler that threads long reads through a de Bruijn graph. In our data it produced highly fragmented assemblies and split plasmid-borne resistance genes across short contigs. OPERA-MS, which scaffolds a short-read backbone with long reads, produced more contiguous assemblies and better MAG quality ratios. That said, OPERA-MS did not always match single-platform long-read contiguity; its scaffolds are limited by the MEGAHIT backbone, which becomes the bottleneck when long-read assemblers resolve complex repeats more effectively.

This echoes isolate genomics, where SPAdes-based hybrid tools like Unicycler often produce more fragmented assemblies than long-read-first approaches (Wick et al., 2017). In metagenomics the problem is worse: a de Bruijn graph containing many related genomes means long reads span conserved regions across different organisms, creating cross-species edges that fragment the graph rather than resolving repeats. The total resistome analysis confirmed this: metaSPAdes hybrid had the lowest plasmid attribution of any strategy in livestock (20–31%) because its short contigs fail geNomad classification, while OPERA-MS reached 27–44%. For mobilome work, scaffolding-based hybrid approaches or single-platform long-read assemblies should be preferred over short-read-centric hybrid methods.

### Practical recommendations

The best platform depends on community diversity. For high-diversity samples (livestock gut, soil, and wastewater), PacBio HiFi should be the primary platform for mobilome work. For lower-diversity communities (human gut, clinical samples), OPERA-MS hybrid assembly may suffice (N50 = 30.8 kb in human). Scaffolding-based hybrid methods (OPERA-MS) should be preferred over de Bruijn graph methods (metaSPAdes hybrid). For MAG recovery from long-read data, SemiBin2 with native coverage outperformed MetaBat2 with Illumina coverage (1.4–2.2× more HQ+MQ MAGs). There is a fundamental trade-off: short reads excel at taxonomic breadth, long reads at structural resolution of mobile elements. A decision flowchart summarizing these recommendations is provided in Fig. S3.

### Limitations

Our study uses three gut communities as case studies for each sequencing platform, and Friedman tests confirmed significant platform effects; however, three samples represent the minimum for this test, and our findings should be validated with larger cohorts before drawing broadly generalizable conclusions. We used a single HMW DNA extraction protocol without mechanical disruption, which was necessary for long reads but may underrepresent Gram-positive organisms compared to bead-beating (Trigodet et al., 2022); this bias affects all platforms equally since the same extraction was used. ONT assemblies were not polished (e.g., with Medaka), which may have contributed to some INTERNAL_STOP artifacts and lower circular plasmid recovery; future studies should incorporate polishing when gene-level accuracy is required. The contig-level MDR plasmid definition inherently favors more contiguous assemblies; although our cross-platform alignment analysis (Table S13) confirmed that the platform gap reflects genuine fragmentation, alternative approaches such as plasmid binning could reduce this bias. Bioinformatic pipelines were simplified to one assembler per platform category and one contig classifier (geNomad); other tools may yield different results, though tool comparison is not the scope of this study. External validation was possible for human gut using paired Illumina/ONT data (Chen et al., 2022), but this validation does not independently confirm PacBio HiFi findings. Paired multi-platform datasets for livestock metagenomes are scarce, limiting external validation for cattle and pig to our downsampling experiment. Despite these caveats, consistent patterns across three taxonomically diverse gut communities support the generality of our findings.

## Figures and Tables

**Fig. 1.**
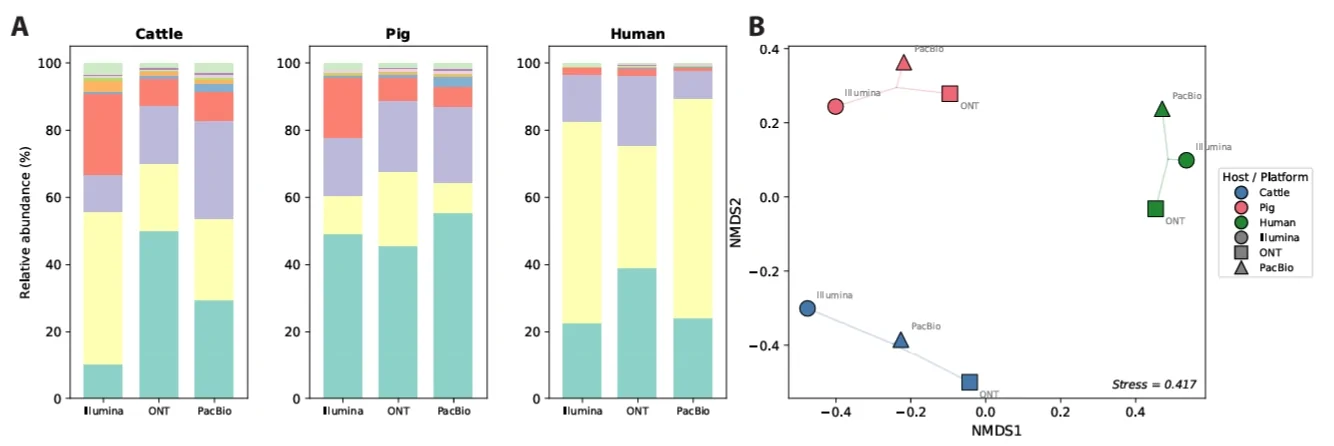
Taxonomic profiling across hosts and platforms. (A) Phylum-level relative abundance based on Kraken2/Bracken classification of quality-filtered reads from cattle, pig, and human samples sequenced on Illumina, ONT, and PacBio HiFi. (B) NMDS ordination of microbial community structure across hosts and platforms (stress = 0.417; values above 0.2 indicate suboptimal two-dimensional fit). ANOSIM confirmed significant clustering by host (R = 1.00, *p* = 0.004) but not by platform (R = −0.23, *p* = 0.872).

**Fig. 2.**
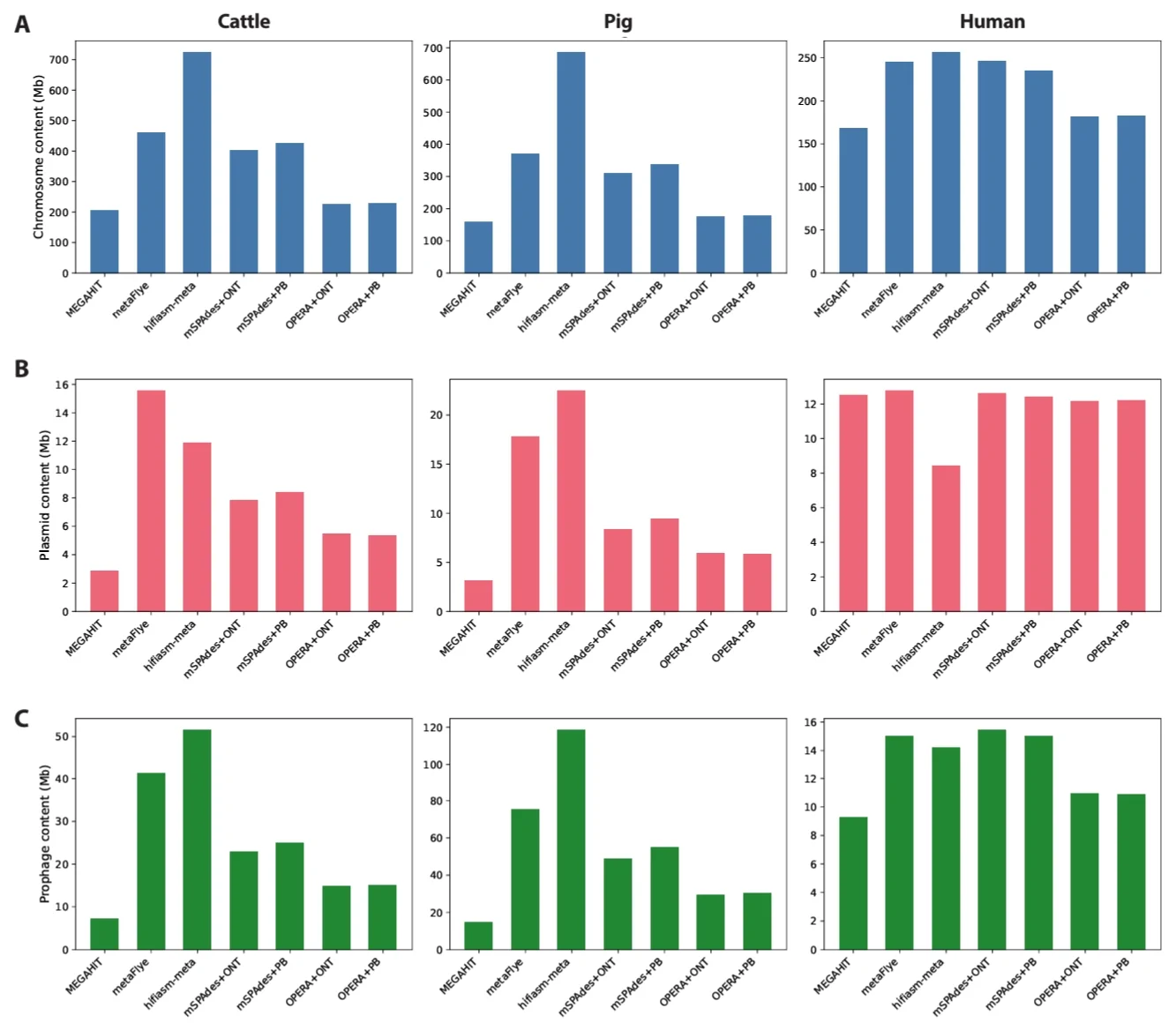
Contig classification across assembly strategies. Total assembled sequence (Mb) classified by geNomad into chromosome, plasmid, and virus categories for seven assembly strategies across cattle, pig, and human gut metagenomes.

**Fig. 3.**
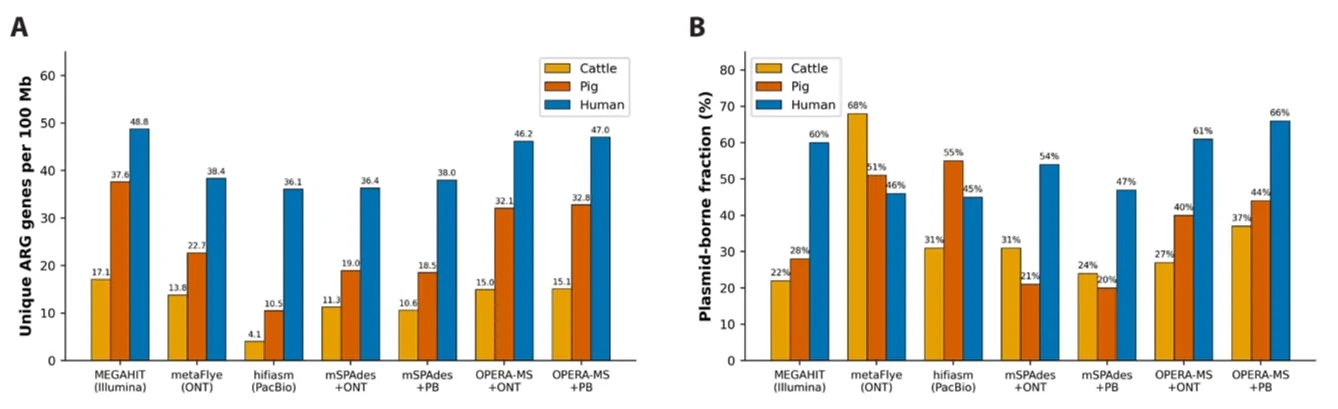
Total resistome comparison across assembly strategies. (A) Unique ARG genes per 100 Mb of assembled sequence. (B) Plasmid-borne fraction of total detected ARGs across cattle, pig, and human samples.

**Fig. 4.**
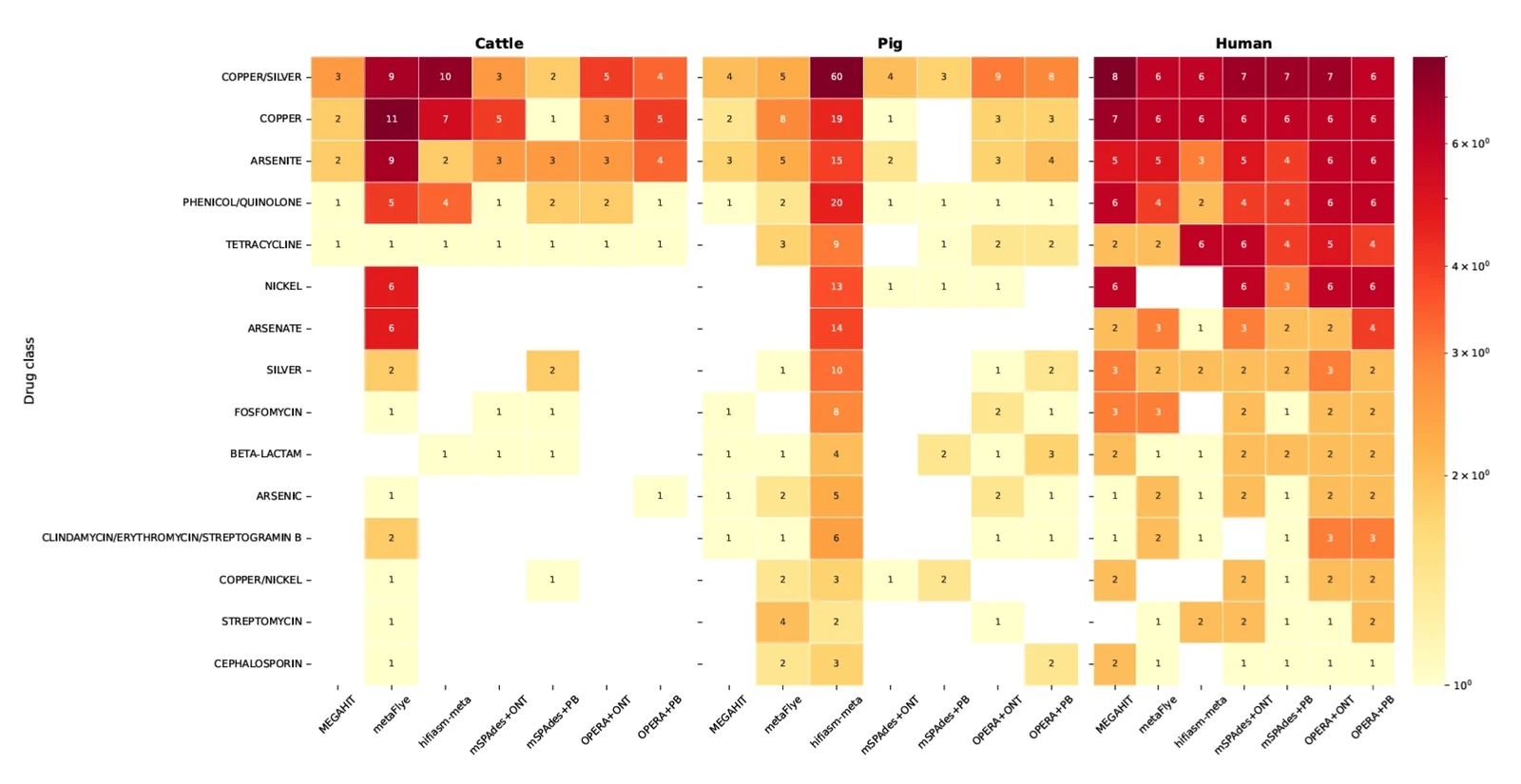
Plasmid-borne ARG detection across assembly strategies. Heatmaps show the number of AMR gene hits for the top drug classes (rows) detected by AMRFinderPlus on geNomad-classified plasmid contigs for each assembly strategy (columns) across cattle, pig, and human samples. Long-read assemblies detect ARGs across more drug classes, particularly metal resistance genes (copper, nickel, arsenic, silver) that are nearly absent from Illumina assemblies. Metal resistance genes are distinguished from antibiotic resistance genes in the Fig.

**Fig. 5.**
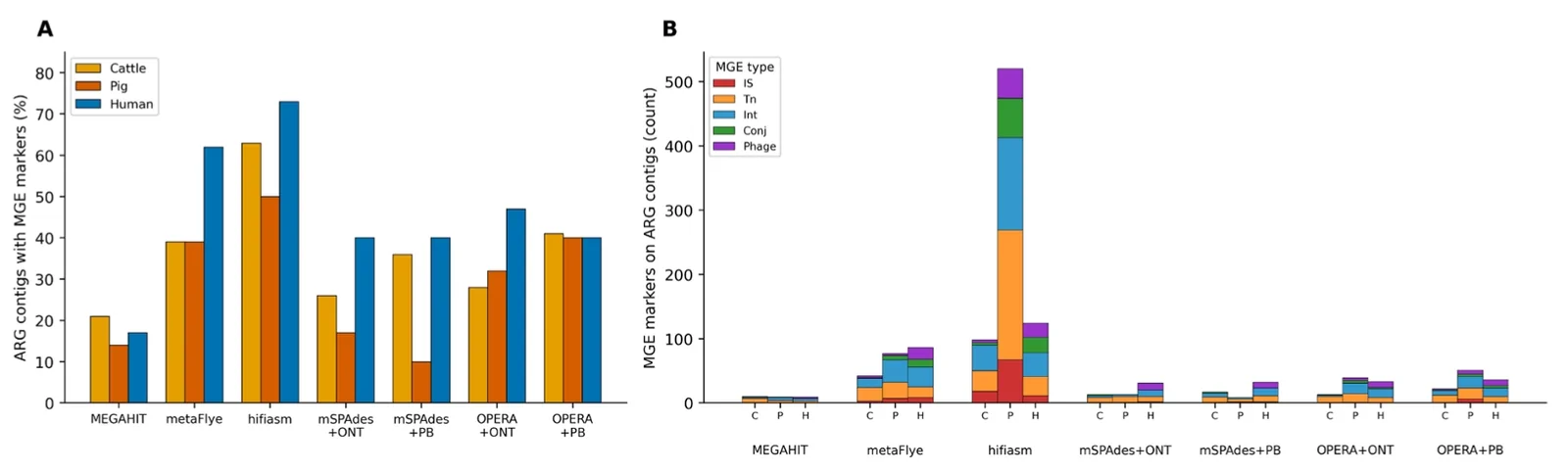
ARG putative mobilization context across assembly strategies. (A) Percentage of ARG-carrying contigs co-located with at least one MGE marker (IS elements, transposons, integrases, conjugation machinery, phage elements). Co-localization suggests a mobilization context but does not confirm actual transferability. (B) MGE type breakdown on ARG contigs across strategies and samples (C, cattle; P, pig; H, human).

**Fig. 6.**
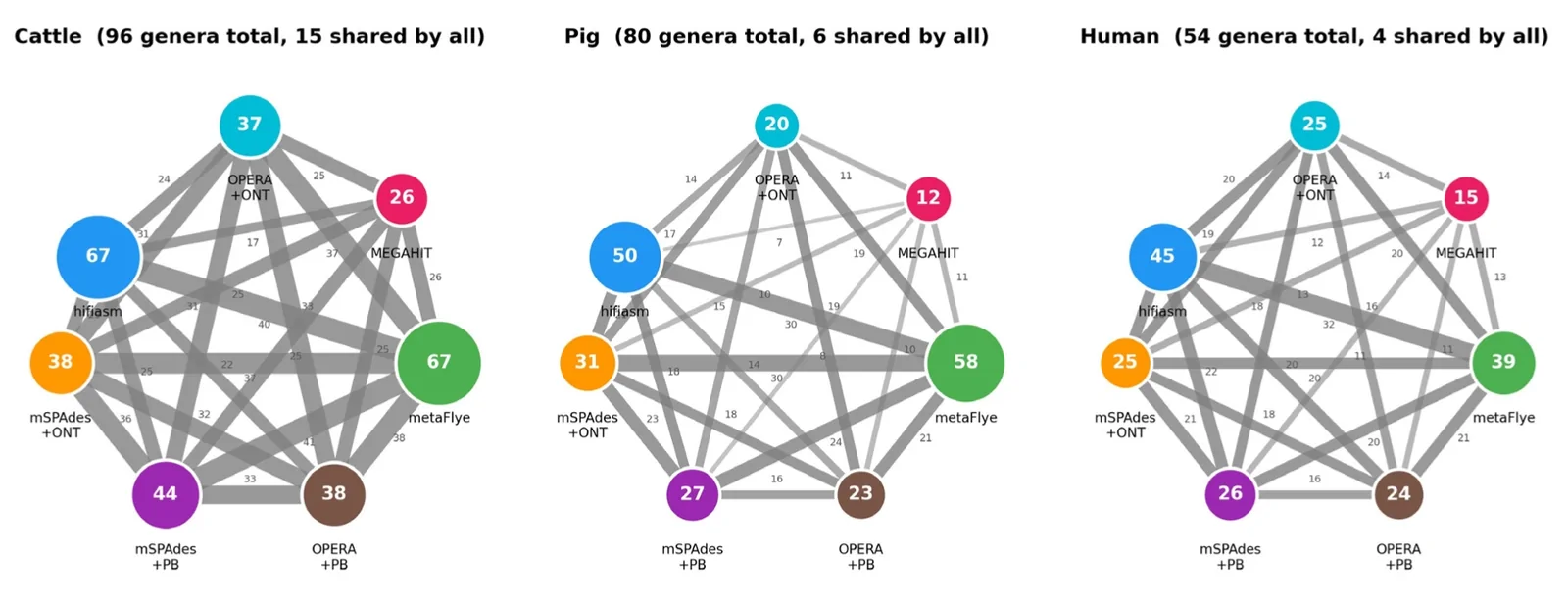
MAG overlap across assembly strategies. Network diagrams show pairwise genus overlap among seven assembly strategies for cattle, pig, and human gut metagenomes. Long-read assemblies were binned with SemiBin2 using native long-read coverage; short-read and hybrid assemblies were binned with MetaBat2 using Illumina coverage. Node size represents the number of unique genera recovered by each strategy (HQ+MQ MAGs); edge thickness is proportional to the number of shared genera between strategy pairs. Numbers on edges indicate shared genus counts. Only 4–16% of genera were shared by all seven strategies, while each strategy recovered unique lineages. metaFlye and hifiasm recovered the most genera across all hosts.

**Table 1.** Plasmid-borne antimicrobial resistance (AMR) gene detection and multi-drug resistance (MDR) plasmid counts across assembly strategies, as identified by AMRFinderPlus on geNomad-classified plasmid contigs. PacBio hifiasm-meta statistics are reported at the subgraph level to avoid inflation from haplotype redundancy.

Sample	Strategy	Total ARG hits	Unique genes	Drug classes	MDR plasmids
Cattle	MEGAHIT	9	8	5	0
	metaFlye	65	49	22	6
	hifiasm	25	10	6	2
	mSPAdes+ONT	18	15	10	2
	mSPAdes+PB	14	12	9	2
	OPERA+ONT	14	10	5	1
	OPERA+PB	16	14	6	1
Pig	MEGAHIT	19	19	13	4
	metaFlye	65	54	29	15
	hifiasm	214	48	28	49
	mSPAdes+ONT	17	15	12	3
	mSPAdes+PB	15	15	11	2
	OPERA+ONT	34	27	18	5
	OPERA+PB	35	31	18	9
Human	MEGAHIT	73	56	18	7
	metaFlye	59	48	22	5
	hifiasm	49	45	18	3
	mSPAdes+ONT	71	54	21	9
	mSPAdes+PB	54	47	18	5
	OPERA+ONT	77	58	21	7
	OPERA+PB	83	64	24	6

**Table 2.** Number and quality distribution of metagenome-assembled genomes (MAGs) recovered by each assembly strategy for the cattle, pig, and human gut metagenomes. Bin quality was assessed with CheckM2, with high-quality (HQ) defined as completeness ≥ 90% and contamination < 5% and medium-quality (MQ) as completeness ≥ 50% and contamination < 10%.

Sample	Strategy	Total bins	HQ (≥ 90C/< 5Ct)	MQ (≥ 50C/< 10Ct)	LQ
Cattle	MEGAHIT	36	16	17	3
	metaFlye	100	32	48	20
	hifiasm	68	9	31	28
	mSPAdes+ONT	60	20	34	6
	mSPAdes+PB	66	23	40	3
	OPERA+ONT	58	30	20	8
	OPERA+PB	60	33	21	6
Pig	MEGAHIT	21	5	10	6
	metaFlye	63	11	35	17
	hifiasm	63	3	24	36
	mSPAdes+ONT	40	4	31	5
	mSPAdes+PB	46	5	31	10
	OPERA+ONT	34	7	21	6
	OPERA+PB	42	7	23	12
Human	MEGAHIT	15	6	9	0
	metaFlye	45	9	25	11
	hifiasm	52	12	23	17
	mSPAdes+ONT	26	13	12	1
	mSPAdes+PB	29	12	14	3
	OPERA+ONT	26	14	11	1
	OPERA+PB	28	9	15	4

## Supplementary Information

Table S1. Sequencing data summary

Table S2. Assembly statistics

Table S3. Taxonomic richness from Kraken2

Table S4. Plasmid contig clustering across platforms

Table S5. Mobilizable ARG analysis

Table S6. Conjugation genes on plasmid contigs

Table S7. Downsampling analysis

Table S8. MAG taxonomy comparison across assembly strategies (MetaBat2 for short-read/hybrid, SemiBin2 for long-read assemblies)

Table S9. Assembly accuracy and annotation quality

Table S10. External validation of human gut plasmid recovery (Chen et al., 2022)

Table S11. ARG density normalized by predicted CDS. Unique ARG counts and ARGs per 1,000 predicted CDS for each assembly strategy and sample, providing an alternative normalization metric less sensitive to assembly size inflation

Table S12. MGE category keyword definitions. Keywords used to classify Prokka product annotations into mobile genetic element categories (IS elements, transposons, integrases/recombinases, conjugation machinery, and phage elements) for the mobilization context analysis

Table S13. Cross-platform alignment of MEGAHIT plasmid contigs to PacBio MDR subgraphs. Minimap2 alignment results showing how many single-class and total MEGAHIT ARG-carrying plasmid contigs map to PacBio MDR plasmid subgraphs, confirming that the MDR plasmid gap reflects genuine fragmentation-driven underdetection

Fig. S1. Plasmid contig length distribution across assembly strategies. Box plots show the distribution of plasmid contig lengths (kb) identified by geNomad for each assembly strategy. Long-read and OPERA-MS hybrid assemblies produced longer plasmid contigs than Illumina-only or metaSPAdes hybrid assemblies.

Fig. S2. Alpha-diversity comparison across host species. Box plots show Shannon diversity index (left) and Chao1 richness (right) for cattle (n = 231), pig (n = 298), and human (n = 322) fecal microbiomes based on 16S rRNA gene sequencing. All pairwise comparisons were significant (*p* < 0.001). Cattle had the highest diversity and richness, followed by pig, then human. Data from Song and Unno (2024).

Fig. S3. Decision flowchart for sequencing platform and assembly strategy selection. The flowchart guides users toward appropriate strategies based on sample complexity (high vs. low microbial diversity), primary research objective (resistome/mobilome profiling, plasmid recovery, MAG reconstruction, taxonomic survey), and resource constraints.
