# Supplementary material for: Long-read sequencing reveals putatively mobilizable resistance genes and multi-drug resistance plasmids underestimated by short-read metagenomics
Source: J Microbiol. 2026 Aug 31;64(8):e2605007. doi: 10.71150/jm.2605007 (PMC13688123; doi:10.71150/jm.2605007)
Supplement: Supplementary file 1 [file jm-2605007-Supplementary-Fig-S1.pdf]

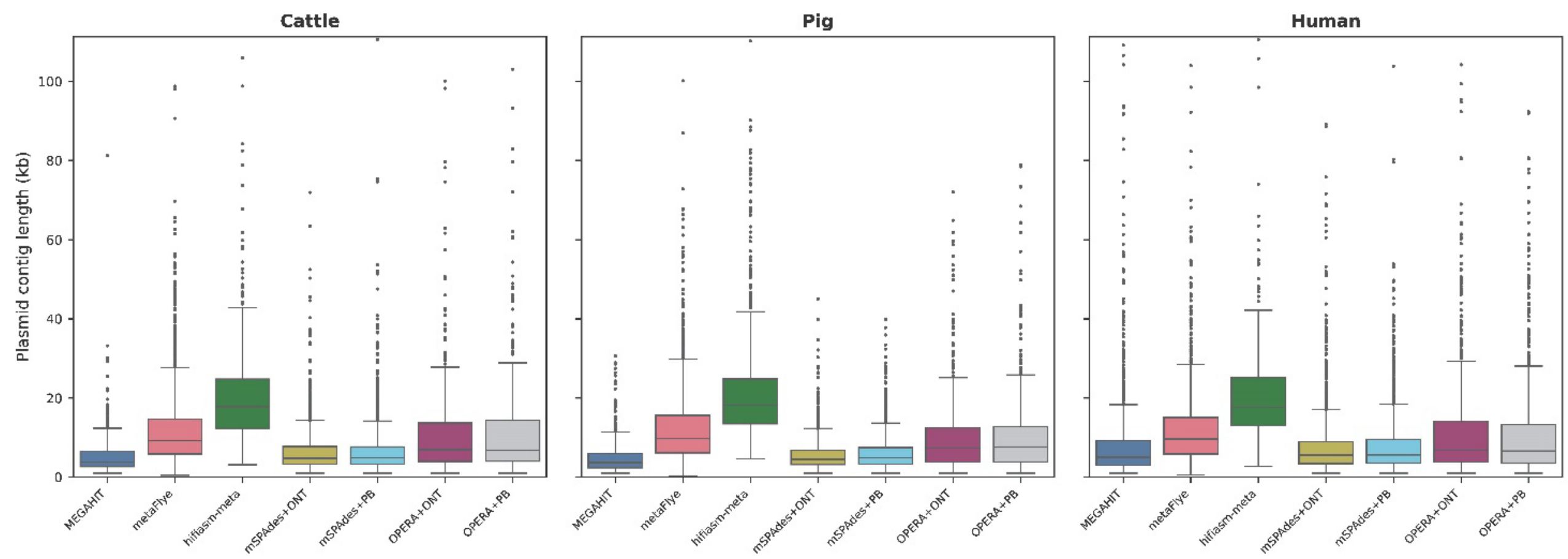

Fig. S1. Plasmid contig length distribution across assembly strategies. Box plots show the distribution of plasmid contig lengths (kb) identified by geNomad for each assembly strategy. Long-read and OPERA-MS hybrid assemblies produced longer plasmid contigs than Illumina-only or metaSPAdes hybrid assemblies.
