# Supplementary material for: Long-read sequencing reveals putatively mobilizable resistance genes and multi-drug resistance plasmids underestimated by short-read metagenomics
Source: J Microbiol. 2026 Aug 31;64(8):e2605007. doi: 10.71150/jm.2605007 (PMC13688123; doi:10.71150/jm.2605007)
Supplement: Supplementary file 2 [file jm-2605007-Supplementary-Fig-S2.pdf]

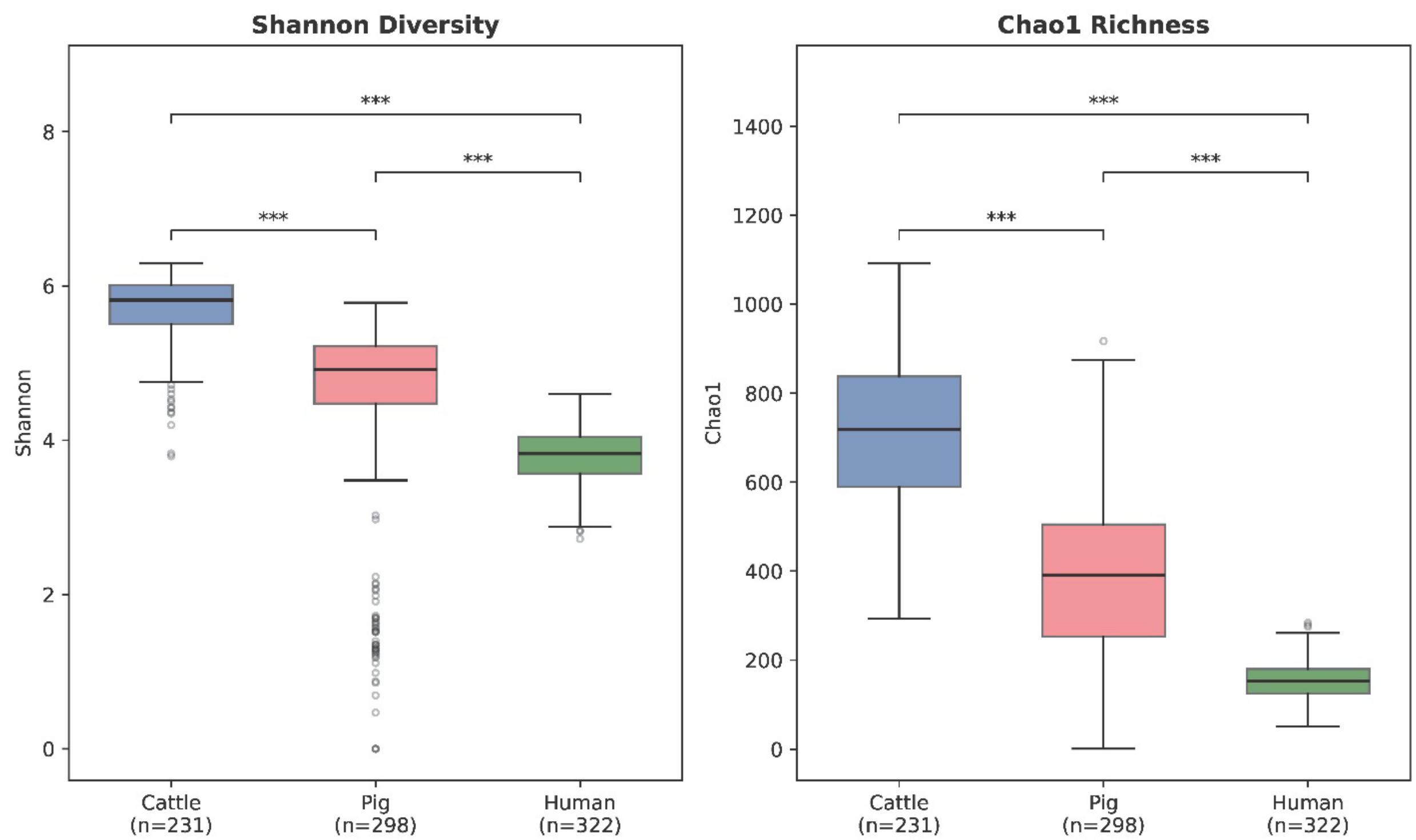

Fig. S2. Alpha-diversity comparison across host species. Box plots show Shannon diversity index (left) and Chao1 richness (right) for cattle (n = 231), pig (n = 298), and human (n = 322) fecal microbiomes based on 16S rRNA gene sequencing. All pairwise comparisons were significant (***, $p < 0.001$). Cattle had the highest diversity and richness, followed by pig, then human. Data from Song and Unno (2024).
