# Supplementary material for: Long-read sequencing reveals putatively mobilizable resistance genes and multi-drug resistance plasmids underestimated by short-read metagenomics
Source: J Microbiol. 2026 Aug 31;64(8):e2605007. doi: 10.71150/jm.2605007 (PMC13688123; doi:10.71150/jm.2605007)
Supplement: Supplementary file 3 [file jm-2605007-Supplementary-Fig-S3.pdf]

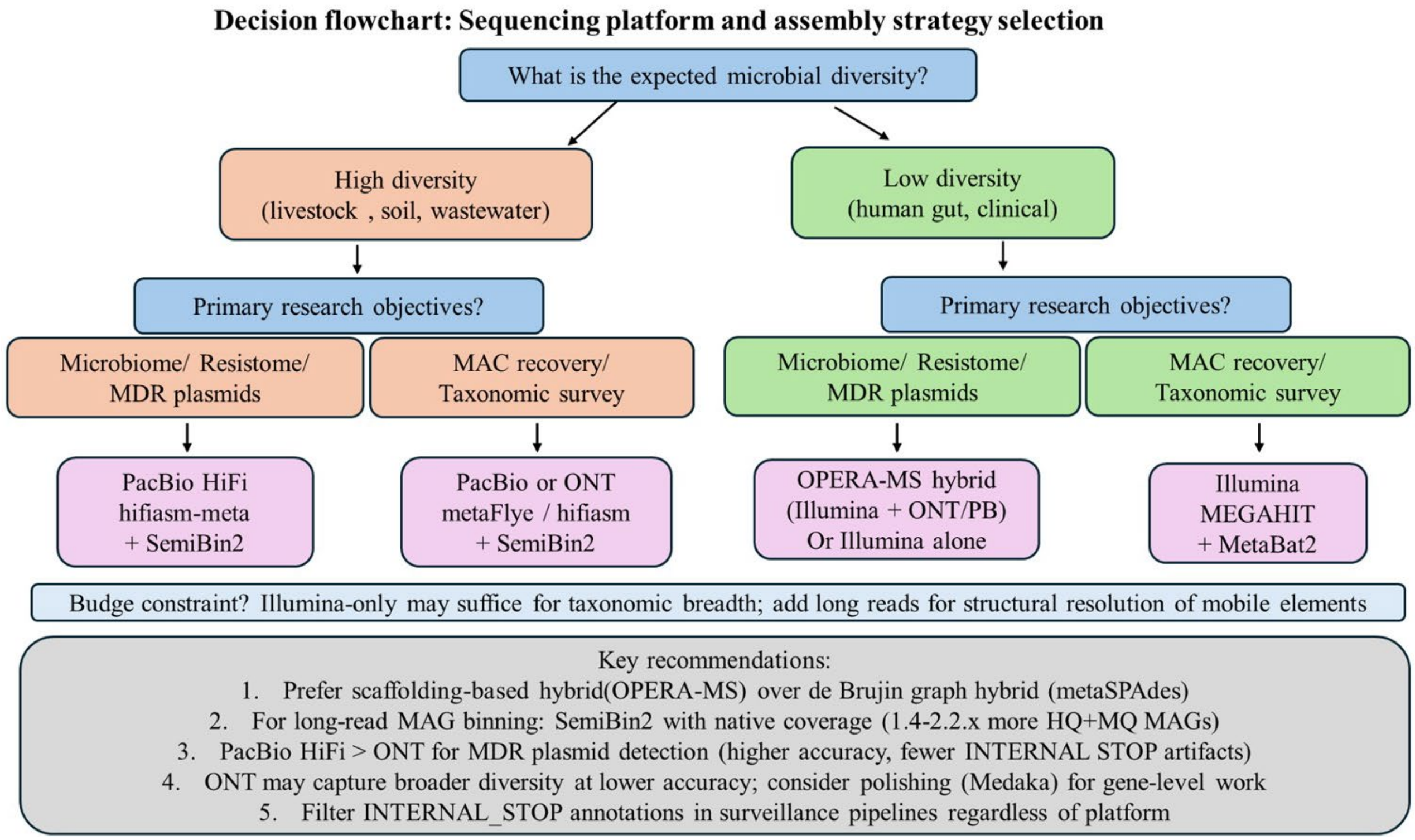

Fig. S3. Decision flowchart for sequencing platform and assembly strategy selection. The flowchart guides users toward appropriate strategies based on sample complexity (high vs. low microbial diversity), primary research objective (resistome/mobilome profiling, plasmid recovery, MAG reconstruction, taxonomic survey), and resource constraints.
